# High *P*_CO_2__ does not alter the thermal plasticity of developing Pacific herring embryos during a marine heatwave

**DOI:** 10.1242/jeb.243501

**Published:** 2022-03-10

**Authors:** Christopher S. Murray, Terrie Klinger

**Affiliations:** School of Marine and Environmental Affairs and Washington Ocean Acidification Center, University of Washington, 3707 Brooklyn Ave NE, Seattle, WA 98105, USA

**Keywords:** Ocean acidification, Extreme climatic events, *Clupea pallasii*, Early life history, Metabolic rates, Embryogenesis, Survival

## Abstract

Forage fish tend to respond strongly to environmental variability and therefore may be particularly sensitive to marine climate stressors. We used controlled laboratory experiments to assess the vulnerability of Pacific herring (*Clupea pallasii*) embryos to the combined effects of high partial pressure of carbon dioxide (*P*_CO_2__) and a simulated marine heatwave. The two *P*_CO_2_ _treatments reflected current conditions (∼550 µatm) and a future extreme level (∼2300 µatm). The dynamics of the heatwave (i.e. rate of onset: ∼0.85°C day^−1^; maximum intensity: +4.4°C) were modeled from the most extreme events detected by a long-term regional temperature dataset. Simultaneous exposure to these potential stressors did not affect embryo survival. However, the heatwave did elicit significant metabolic effects that included higher rates of routine metabolism (*Q*_10_=1.15–1.72), growth (*Q*_10_=1.87), rate of development to hatch (*Q*_10_=3.01) and yolk consumption (*Q*_10_=3.21), as well as a significant reduction in production efficiency (−10.8%) and a three-fold increase in the rate of developmental anomalies. By contrast, high *P*_CO_2__ conditions produced comparatively small effects on vital rates, including a significant increase in time to hatch (+0.88 days) and a reduction in routine metabolic rate (−6.3%) under the ambient temperature regime only. We found no evidence that high *P*_CO_2__ increased routine metabolic rate at either temperature. These results indicate that Pacific herring embryos possess sufficient physiological plasticity to cope with extreme seawater acidification under optimal and heatwave temperature conditions, although lingering metabolic inefficiencies induced by the heatwave may lead to important carryover effects in later life stages.

## Abstract (precis)

**Summary:** Pacific herring embryos tolerated exposure to a simulated marine heatwave, but exhibited significant metabolic trade-offs. Exposure to high *P*_CO_2__ induced few metabolic effects and did not alter thermal plasticity.

## INTRODUCTION

Extreme climatic events, in which environmental conditions rapidly shift beyond predictable variability, pose an immediate threat to ecological communities (Frölicher et al., 2018; Wernberg et al., 2013; Wetz and Yoskowitz, 2013). Phenotypic plasticity may allow individual organisms to withstand periods of extreme stress by adjusting their phenotype through changes in gene expression or function to match the new environment (Fox et al., 2019; Seebacher et al., 2015). However, the capacity for phenotypic plasticity is inherently limited, and thus successful acclimatization will depend on the specific attributes of the extreme event (i.e. the rate of onset, intensity and duration). This situation is complicated further by the fact that plasticity can vary with ontogeny such that specific life stages face a heightened vulnerability to potential stressors (Burggren, 2018). For example, marine fish display a range of plastic responses in behavior, morphology, phenology and physiology that enable species and populations to persist across large spatial and temporal environmental gradients (Jonsson and Jonsson, 2019). However, fish embryos lack the robust homeostatic capacity that is enabled by mature organ systems and must rely largely on molecular mechanisms rather than behavioral plasticity to counter stressful conditions (Dahlke et al., 2020b; Melzner et al., 2009). Indeed, the early life stages of many fish taxa constitute a critical sensitivity bottleneck in the face of emerging climate change (Baumann, 2019; Esbaugh, 2018; Pörtner and Peck, 2010).

Given that the thermal biology of fish embryos has been studied for decades, there are clear expectations regarding how physiological processes will respond to warming. For example, we know that embryos of temperate fish species have a very limited capacity to regulate metabolic rate against acute temperature change [across-species mean temperature coefficient (*Q*_10_)≈3.0] and consequently embryos exhibit roughly half the thermal tolerance range compared with juveniles and non-spawning adults of the same species (Rombough, 2011, 1997). By contrast, the extent to which embryonic metabolism is affected by elevated partial pressures of carbon dioxide (*P*_CO_2__) consistent with near-future ocean acidification (OA) is less understood. Embryos with underdeveloped gills and circulatory systems rely predominately on intracellular mechanisms of pH control (Brauner et al., 2019; Mölich and Heisler, 2005). Acid–base perturbations can be responded to through the increased expression of membrane-bound ion pumps, transporters and gas channels (Brauner et al., 2019; Talbot et al., 2015; Tseng et al., 2013). Acidification also triggers the upregulation of stress and repair genes, including heat shock proteins and enzymes involved in antioxidant pathways (Tiedke et al., 2013). Maintaining these functions under elevated *P*_CO_2__ can be energetically costly and observations of isolated fish tissues have shown that acidification triggers higher metabolic rates (Heuer and Grosell, 2016; Kreiss et al., 2015). If embryos allocate their fixed energetic reserves in a compensatory manner, then the redirection of energy towards acid–base homeostasis should come at the expense of critical growth and development (Rombough, 1994). Furthermore, acidification could limit thermal acclimation because the additional energetic costs of acid–base regulation could reduce the pool of energy available to support higher metabolic rates during acute warming (Pörtner, 2008).

To date, very few studies have performed respiratory measurements on fish embryos to test for metabolic shifts under elevated *P*_CO_2__ conditions consistent with OA. Atlantic cod (*Gadus morhua*) and polar cod (*Boreogadus saida*) showed an ∼10% increase in respiration rates when exposed to 1100 µatm *P*_CO_2__ across most of the respective temperature ranges, except for extreme high temperatures, where the effect was reversed and elevated *P*_CO_2__ induced a 10–20% reduction to rates of oxygen consumption (Dahlke et al., 2018, 2017). The authors hypothesized that this metabolic response to combined stressors was the result of reduced mitochondrial capacity to form ATP. In all cases, elevated *P*_CO_2__ acted to reduce the size of newly hatched cod by 10–13%. By contrast, elevated *P*_CO_2__ had no effect on embryonic metabolism or growth across relevant temperature ranges in the Antarctic dragonfish (*Gymnodraco acuticeps*; Flynn et al., 2015), Atlantic herring (*Clupea harengus*; Leo et al., 2018) and Atlantic silverside (*Menidia menidia*; Murray and Baumann, 2018; Schwemmer et al., 2020). Embryos of the ocellated wrasse (*Symphodus ocellatus*) that were transplanted into a high *P*_CO_2__ environment adjacent to a volcanic CO_2_ seep showed an ∼50% increase in respiration, whereas embryos sourced from a population endemic to the CO_2_ seeps showed no effect (Cattano et al., 2016). Thus, local adaptation to prevailing *P*_CO_2__ conditions or multigenerational plasticity could help shape CO_2_ reaction norms among offspring (Murray et al., 2014). Together, these studies suggest that elevated levels of *P*_CO_2__ consistent with future acidification (1000–2000 µatm) will produce neutral to perhaps moderate (<±20%) changes to the metabolic rates of fish embryos. Yet, the paucity of studies and species tested to date limits the ability to predict responses in unexamined taxa, particularly when combined with extreme temperature variability.

In the Pacific Northwest (USA), marine ecosystems are under threat from the major climate-associated stressors, including OA and extreme heatwave events (Mauger et al., 2015). The Salish Sea ecosystem is particularly vulnerable to OA owing to a confluence of anthropogenic and natural factors that already cause seasonal *P*_CO_2__ levels to regularly exceed those shown to be stressful for some marine ectotherms (Evans et al., 2019; Fassbender et al., 2018; Feely et al., 2010), and these will worsen in the future (Pacella et al., 2018). At the same time, the region has recently experienced a series of marine heatwave events, defined as prolonged periods of anomalously warm ocean temperatures that are quantitatively distinct in time and space (Hobday et al., 2016). The North Pacific marine heatwave of 2014–2016 was among the most extreme events on record, during which positive temperature anomalies of 3–6°C persisted for months at a time over much of the northeast Pacific (Bond et al., 2015; Di Lorenzo and Mantua, 2016; Gentemann et al., 2017). The ecological toll of this event was considerable as effects were felt at all trophic levels (Fisher et al., 2020; Piatt et al., 2020; von Biela et al., 2019). Nearshore habitats face the compounding threat of intense atmospheric heatwaves that can rapidly warm shallow systems over the course of hours to days, with lethal consequences for nearshore organisms (Schlegel et al., 2017; Thomsen et al., 2019). For most coastal regions, the frequency of acute nearshore heatwaves remains largely unquantified and the potential for interactions with large-scale marine heatwaves is complex and poorly described, which limits our ability to anticipate how severe these events might become in the immediate future (Schlegel et al., 2017).

Forage fish dominate the mid-trophic levels of many coastal marine ecosystems and serve as a critical conduit of biological production from lower trophic levels to higher order predators (Pikitch et al., 2012). The life history characteristics of many forage fishes lend to ‘boom or bust’ population dynamics because recruitment processes are heavily influenced by environmental variability (Szuwalski et al., 2019; Szuwalski and Hilborn, 2015). Consequently, the future viability of forage fish stocks is complicated by marine climate change (Merino et al., 2010; Shannon et al., 2009). The Pacific herring (*Clupea pallasii*) occupies a central position in the Salish Sea food web, and all life stages serve as important prey items for a multitude of marine taxa, including sea birds, mammals, and commercially important fish and invertebrates (Penttila, 2007). Despite reduced fishing pressure and intensive conservation efforts, the spawning stock biomass of several Salish Sea herring populations has declined in recent decades (Sandell et al., 2019; Siple and Francis, 2016; Siple et al., 2017). Understanding how extreme environmental variability will influence herring recruitment processes is a question of ecological and socio-economic importance.

Pacific herring reproduce in shallow nearshore environments where adults adhere fertilized embryos to submerged vegetation (Penttila, 2007; Shelton et al., 2014). In the Salish Sea, these nearshore systems have been modified by human activities (Penttila, 2007) and are exposed to episodic events of low seawater pH associated with sharp fluctuations in *P*_CO_2__ (Evans et al., 2019; Pacella et al., 2018). Spawning phenology is tightly correlated with photoperiod (Petrou et al., 2021), and 20 of the 21 spawning populations found in the southern Salish Sea reproduce from January to April, when water temperatures are generally below 11°C, with the exception of the Cherry Point stock, which reaches peak reproductive activity in late May (Hay, 1985; Sandell et al., 2019). Decades of empirical research have demonstrated that the viability of Pacific herring embryos is maximized between 8 and 10°C and quickly declines with increasing temperature (Alderdice and Hourston, 1985; Alderdice and Velsen, 1971; Dinnel et al., 2010). In the Salish Sea, nearshore heatwaves occur during all seasons (see Supplementary Materials and Methods) and severe acidification events frequently occur during winter and spring (Evans et al., 2019). The simultaneous emergence of these potential stressors shortly after a spawning event could compromise the viability of entire cohorts of newly fertilized embryos.

Clupeid offspring have been shown to be sensitive to the combined effects of elevated *P*_CO_2__ and temperature through changes in survival and development (Franke and Clemmesen, 2011; Frommel et al., 2014; Leo et al., 2018; Sswat et al., 2018; Villalobos et al., 2020). Yet, the phenotypic plasticity of Pacific herring embryos exposed to combined climate stressors remains understudied, thereby constituting a serious knowledge gap for this critically important species. Accordingly, we used laboratory experiments to test the hypothesis that a heatwave event will reduce embryo survival and metabolic efficiency and that high *P*_CO_2__ conditions will aggravate physiological effects by (1) increasing the energetic requirements of acid–base regulation while (2) simultaneously limiting the pool of energy that is available for effective thermal acclimation.

**List of symbols and abbreviations**

*A*: assimilation
*A*_T_: total alkalinity
CI: confidence interval
CRM: certified reference material
ddpf: degree-days post-fertilization
DIC: dissolved inorganic carbon
dpf: days post-fertilization
dph: days post-hatch
*E*: excretion
hpf: hours post-fertilization
LMM: linear mixed model
*Ṁ*_O_2__: oxygen consumption rate
NERRS: National Estuarine Research Reserve System
OA: ocean acidification
*P*: production
PCA: principal components analysis
*P*_CO_2__: partial pressure of carbon dioxide
pH_NIST_: pH on the National Institute of Standards and Technology scale
pH_T_: pH on the total scale
*Q*_10_: temperature coefficient
*R*: respiration
RMR: routine metabolic rate

## MATERIALS AND METHODS

### Collection and description of spawning adults

Animal care and experimental procedures followed the guidelines set forth by the Animal Care Office of Western Washington University (protocol no. 20-002). Wild *Clupea pallasii* Valenciennes in Cuvier and Valenciennes 1847 that were ripe for spawning were collected from Semiahmoo Bay, WA, USA, on 19 February 2020 by the Washington Department of Fish and Wildlife. The Semiahmoo Bay population is a winter-spawning stock that reproduces during February through early March (Sandell et al., 2019). Despite considerable spatial overlap with the spring-spawning Cherry Point stock, the two populations remain genetically isolated as a result of differing phenological patterns (Petrou et al., 2021; Small et al., 2005). The Semiahmoo Bay population does retain considerable genetic exchange with the other winter-spawning stocks from the Salish Sea (Small et al., 2005). Gonads were dissected from nine females and three males and were stored in glass dishes covered with moist paper towels at 4.5°C for 48 h (Dinnel et al., 2010). The mean (±s.d.) total length, wet mass and gonadic–somatic index of female and male spawners was 18.4±1.0 and 19.7±1.3 cm, 51.9±7.3 and 59.6±13.9 g, and 19.5±3.0 and 17.2±1.7%, respectively.

### Criteria for treatment *P*_CO_2__ and temperature conditions

**Fig. 1.**
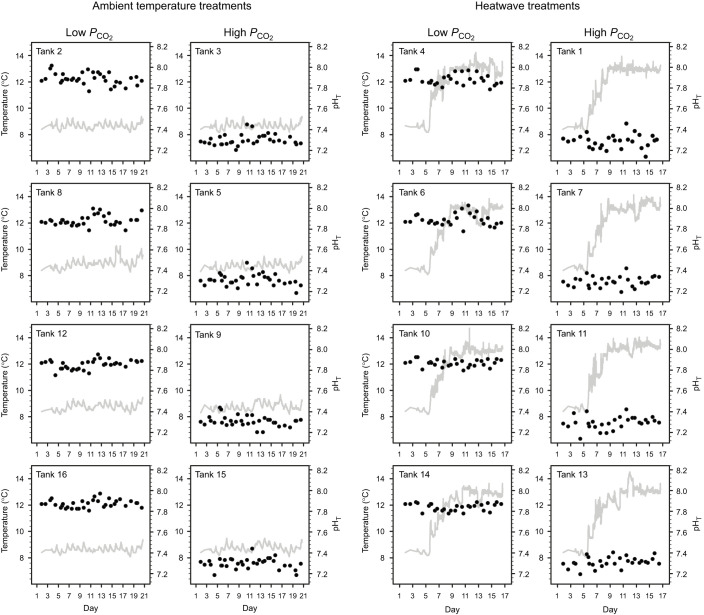
**Treatment conditions by replicate tank.** Continuous temperature measurements (gray lines) and daily spot-checks of pH on the total scale (pH_T_; black circles) in 16 replicate tanks. Note that the experimental trials were terminated at the end of the hatching period (heatwave: 17 dpf; ambient temperature 21 dpf).

The experiment was conducted at Western Washington University's Shannon Point Marine Center (Anacortes, WA, USA) using 16 purpose-built experimental flow-through units. Embryos were reared under two *P*_CO_2__ levels [low *P*_CO_2__: ∼550 µatm, pH on the total scale (pH_T_) 7.88; elevated *P*_CO_2__: ∼2300 µatm, pH_T_ 7.30] crossed with two temperature regimes (ambient and heatwave). The low *P*_CO_2__ treatment is representative of surface seawater conditions for the Salish Sea during late winter (Fassbender et al., 2018), whereas the high *P*_CO_2__ treatment reflects maximum of end-of-century *P*_CO_2__ predicted for the coastal ecosystems of the Pacific Northwest (Evans et al., 2019; Pacella et al., 2018). The ambient temperature treatment fluctuated with the natural conditions (average: 8.8±0.3°C, range: 8.1–10.4°C; Fig. 1) at the laboratory's seawater source (Guemes Channel, 48°30′34.5″N 122°41′04.5″W), which is near the optimal temperature for embryonic development of Salish Sea Pacific herring (Alderdice and Velsen, 1971; Dinnel et al., 2010). All embryos were reared at ambient temperature conditions until ∼3.5 days post-fertilization (dpf) or approximately one-quarter of the embryonic duration for *C. pallasii* at ∼8.8°C (Kawakami et al., 2011). Heatwave conditions were initiated 88 h post-fertilization (hpf) in half of the replicate tanks per *P*_CO_2__ level (treatment *N*=4). Temperatures were increased by ∼0.85°C day^‒1^ for five consecutive days to achieve a heatwave condition of +4.4°C above ambient (∼8.8°C to ∼13.2°C), which was maintained for the duration of the experiment (Fig. 1).

Heatwave characteristics (i.e. rate of onset, duration and maximum intensity) were determined by analyzing a 19-year temperature dataset from the nearby NERRS Ploeg Channel monitoring station in Padilla Bay, WA [48°33′22.8″N 122°31′51.2″W; http://www.nerrsdata.org (accessed 2 November 2019)]. The station monitors a channel that drains a network of eelgrass beds and tidal flats and thus conditions are broadly representative of herring spawning habitat in the Salish Sea (Bulthuis, 2013). The R package heatwaveR (Schlegel and Smit, 2018) was used to identify heatwaves in the time series based on selection criteria of daily mean temperatures exceeding the 90th percentile of the long-term seasonal climatology for a minimum of five consecutive days (Hobday et al., 2016). The experimental heatwave treatment was based on the average characteristics of the top five heatwave events on record corresponding to a category 2 heatwave as defined by Hobday et al. (2018). See Supplementary Materials and Methods for complete details. The viability of Pacific herring embryos begins to decline from optimal as temperature exceeds 10.5°C and embryonic viability rapidly declines when incubation conditions climb above 13°C (Fig. S1; Alderdice and Velsen, 1971; Dinnel et al., 2010). Thus, the maximum temperature of the simulated heatwave event may approach critical temperature thresholds where interactive effects with high *P*_CO_2__ are hypothesized to emerge (Lefevre, 2016; Pörtner, 2010).

### Experimental system

The 16 experimental units were each composed of an elevated 40-liter mixing tank and a 12-liter main rearing tank. The mixing tank received a continuous supply (∼1 l min^−1^) of filtered (to 1 µm) seawater (salinity ∼32 ppt) with a stable pH_T_ of ∼7.66. To achieve high *P*_CO_2__ conditions, the mixing tank continuously received standardized doses of CO_2_ gas (99% CO_2_, AirGas) delivered by an eight-channel peristaltic pump (Masterfelx L/S, Cole-Parmer). CO_2_ was injected directly into the intake of a submersible pump with magnetically driven impellers to promote an even dissolution. Low *P*_CO_2__ conditions were achieved by stripping CO_2_ from compressed air (CO_2_ adsorber, Twin Tower Engineering), which was then bubbled directly into mixing tanks to purge dissolved inorganic carbon (DIC) from seawater. All mixing tanks were continuously bubbled with compressed laboratory air to maintain a dissolved oxygen partial pressure (*P*_O_2__)>85% air saturation. Heatwave temperatures were controlled by Elitech STC-1000 controllers that powered three 100 W heaters (Aqueon) per unit. During heatwave onset, temperature set points were increased by 0.5°C every 12 h. Small aquarium pumps were positioned in the main rearing tanks to ensure even heating.

Experimental embryos were housed in customized 0.5-liter polyethylene rearing baskets fitted with 300-µm mesh bottoms. Baskets were floated in the main rearing tank. Treated seawater from the mixing tanks was gravity-fed directly into baskets at standardized rates (∼0.5 ml s^−1^). The temperatures of main rearing tanks were recorded automatically every 30 min starting at 3 dpf (HOBO^®^ Pendant Temp, Onset). Embryo baskets were measured for pH and temperature every ∼12 h by a handheld pH meter (Orion Star A221 meter, 9107BNMD pH triode, Thermo Fisher Scientific) which was calibrated daily with three-point NIST buffers (Fisher Chemical). The pH electrode was cleaned daily in 0.1 mol l^−1^ HCl and the reference solution was completely exchanged every 5 days. Measured pH_NIST_ values were converted to pH_T_ via a probe-specific conversion formula (see Supplementary Materials and Methods). Dissolved oxygen levels were checked daily with a handheld meter (YSI Model 55) that was calibrated daily using 100% water-saturated air. Embryos were exposed to dim light over a natural light cycle (11 h:13 h light:dark). Experimental pH and temperature conditions are summarized in Fig. 1.

### Carbonate chemistry

Discrete seawater samples (25 ml) were taken every third day from each replicate tank (*n*=5 samples per heatwave replicate; *n*=6 samples per ambient replicate) for direct measurements of pH_T_ and DIC following methods described by Love et al. (2017). Temperature, pH_NIST_ and salinity were recorded at the time of sampling using the handheld electrode. The samples were filtered to 0.6 µm, poisoned with 10 µl of saturated mercuric chloride solution and stored in 25-ml scintillation vials at 2°C. Restricted access to laboratory facilities during the spring of 2020 led to an unplanned and prolonged delay in sample processing (∼4 months) during which significant off-gassing of dissolved CO_2_ from high *P*_CO_2__ samples occurred. Therefore, additional steps were required to estimate carbonate chemistry parameters.

A DIC analyzer (Apollo SciTech AS-C3) was calibrated by developing a standard curve based on five measurements of different volumes (0.5–1.5 ml) of certified reference material (CRM; batch no. 169; Andrew Dickson, University of California San Diego, Scripps Institution of Oceanography, https://www.nodc.noaa.gov/ocads/oceans/Dickson_CRM/batches.html). To measure DIC, samples were warmed to room temperature and the analyzer extracted a minimum of three 1-ml measurements per sample, consisting of a blank followed by at least two consecutive duplicate measurements within 2 μmol l^−1^. DIC was converted to μmol kg^−1^ using the sample density based on measurement room temperature and sample salinity. Seawater samples were analyzed concurrently for pH_T_ using a diode array spectrophotometer (Agilent 8453A UV-VIS). Seawater was injected by syringe into a 5-cm jacketed cuvette that was maintained at room temperature via circulated water from a thermostat bath. After the cuvette was loaded into position and the spectrophotometer was blanked, two 30-µl aliquots of *m*-cresol dye (lot no. MKBR3556V) were injected into the cuvette to generate ‘two-shot’ duplicate measurements of absorbance at three wavelengths (730, 578 and 434 nm). Calculations for pH_T_ were based on absorbance ratios by following Dickson et al. (2007) and included a lot-specific dye correction.

CO_2_ off-gassing meant that measured DIC and pH_T_ could not be directly used to estimate treatment *P*_CO_2__ conditions. However, the measurements were sufficient for estimating *in situ* total alkalinity (*A*_T_, µmol kg^−1^) at the time of sampling given that the *A*_T_ of poisoned samples is unaffected by gas exchange and will closely reflect conditions at the time of sampling in the absence of biological activity (Dickson et al., 2007). Sample *A*_T_ was calculated using the R package seacarb (https://cran.r-project.org/package=seacarb) based on direct measurements of salinity, DIC, pH_T_, typical nutrient levels for the seawater source (total P: 2.2 µmol kg^−1^; total Si: 2 µmol kg^−1^), and the room temperature during DIC and pH_T_ measurements. Equilibrium constants for the dissociation of carbonic acid in seawater (K1 and K2) followed Mehrbach et al. (1973) refitted by Dickson and Millero (1987) and the constant for KHSO_4_ from Dickson (1990). This method was able to consistently reconstruct the *A*_T_ of Dickson CRMs within ±1% and derived *A*_T_ values closely matched a regional salinity and alkalinity relationship (+2% on average; Fassbender et al., 2016).

The remaining *in situ* seawater chemistry parameters of *P*_CO_2__, DIC and carbonate and bicarbonate ion concentrations (CO_3_^−2^, HCO_3_^−^; μmol kg^−1^) were calculated in seacarb using derived *A*_T_, typical nutrient levels, and the *in situ* salinity, temperature and pH_NIST_ levels that were recorded at the time of sampling during the experiment. To reduce the error associated with seawater pH measurements calibrated on the NIST scale (Dickson et al., 2015), an extensive cross-calibration between the handheld electrode and the diode array spectrophotometer was conducted to produce a robust probe-specific linear relationship to convert pH_NIST_ values to pH_T_ (*N*=36, *R*^2^=0.997; see Supplementary Materials and Methods for complete details):
(1)

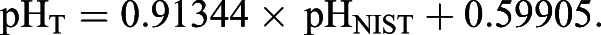


All seacarb calculations used converted pH_T_ values. A summary of treatment carbon chemistry is listed in Table 1.

**Table 1.**
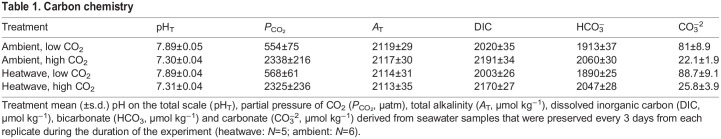
Carbon chemistry

### Fertilization protocols

Embryos were fertilized on 21 February 2020 following established protocols (Dinnel et al., 2010). Gonads were slowly warmed in a walk-in environmental chamber to the fertilization temperature (8.5°C). Four large plastic dishes (two per *P*_CO_2__ treatment) were lined with 300-μm nylon mesh screening and then were filled with 750 ml of filtered and autoclaved seawater (salinity 32) that was adjusted to 550 or 2300 µatm *P*_CO_2__. Five 1-cm^2^ sections of teste were sampled from each male and were eviscerated together on a 300 µm screen and the milt was filtered into a glass beaker filled with 1 liter of clean seawater. The milt solution was mixed and 250 ml was poured into each spawning dish. Small groups of healthy eggs from a single female were then scooped sequentially into the spawning dishes such that eggs were equally but randomly distributed to the four dishes. This process continued with each female until the spawning screens were largely covered with a single layer of eggs. Gametes were left to soak for 1 h after which the screens were rinsed with clean seawater to remove milt and unattached eggs. Adhered embryos were disinfected for 10 min in a 100 ppm Ovadine–seawater solution (Western Chemical). Screens were haphazardly cut into small sections and hung within the floating embryo baskets in each of the four replicate tanks per treatment.

### Sampling design and response variables

#### Fertilization success

Embryos were randomly subsampled (*n*>20) at 46 hpf from each replicate and photographed using a digital camera (MC170 HD, Leica) mounted on a dissection microscope (SZ40 stereomicroscope, Olympus) to produce calibrated digital images (Leica Application Suite). Fertilization status was determined by the presence or absence of a raised fertilization membrane (Dinnel et al., 2010). Fertilization success was quantified as the ratio of fertilized eggs to the total number of eggs visible in the frame.

#### Embryo survival

At exactly 2 dpf, 100 fertilized embryos were randomly selected and distributed to a new rearing basket to quantify survival rates to hatching. The remainder of the embryos were maintained in a separate basket for measurements of developmental and metabolic rates. An accident compromised an embryo survival replicate (tank 1; heatwave×high *P*_CO_2__) and it was removed from the experiment. Baskets were inspected at noon of each day and newly hatched larvae were counted, removed and evaluated for obvious developmental anomalies including hatchlings that exhibited notochord twists, shortenings and curvatures that exceeded 90 deg as well as edemas present in the yolk sac and underdeveloped jaws (Dinnel et al., 2010; Hershberger et al., 2005; Purcell et al., 1990). See Fig. S2 for examples of anomalies. The experimental trials were terminated after three consecutive days of no hatching within each temperature group, which occurred at 17 and 21 dpf under heatwave and ambient temperatures, respectively. The remaining embryos were counted and inspected for developmental status and all unhatched individuals were confirmed to be dead. Total hatch (%) was calculated as the number of hatched larvae (including anomalies) divided by the number of embryos at experiment initiation (*n*=100). Anomaly rate (%) was calculated as the number of anomalous larvae per 100 initial embryos. Hatching success (%) was calculated as the total number of hatched embryos minus the number of anomalous larvae divided by 100 initial embryos. Time to peak hatch was defined as the dpf to 50 cumulative hatchlings. A developmental rate (% day^−1^) was calculated for each replicate as 100% divided by dpf to peak hatch.

#### Metabolic rate

To estimate treatment effects on the routine metabolic rate (RMR) of pre-absorptive and un-anesthetized embryos (Peck and Moyano, 2016), closed respirometry was used to measure the oxygen consumption rates (*Ṁ*_O_2__) of groups of embryos from each replicate tank once per day for three consecutive developmental days. Measurements were initiated at 6 dpf on eye-staged embryos (stage F–G; Kawakami et al., 2011) and then were repeated on 7 and 8 dpf. This period coincided with days 2–4 of the onset of the heatwave. Twenty identical respiration vials were prepared for the experiment: 2.15-ml glass vials fitted at the bottom with a single planar trace oxygen sensor spot (PreSens). All vials were enclosed with screw-tight caps and PTFE/silicone/PTFE septa. Respirometry trials were run separately for each temperature treatment on each developmental day such that six total trials were completed. On the day of sampling, groups of 9 or 10 embryos were randomly selected per replicate tank and disinfected in a 100-ppm Ovadine–seawater solution to reduce background microbial respiration. Post-disinfection, embryos were evaluated under a dissection microscope for physical condition and photographed for calibrated measurements of egg surface area (mm^2^) and diameter cross-sections from three different axes (mm). Egg volume (*V*; μl) was estimated from diameter measurements using the ellipsoid formula:
(2)

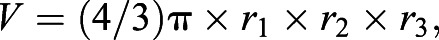
where *r* is radius. All 9–10 embryos per replicate tank were transferred into a single randomly selected respiration vial filled with UV-sterilized and autoclaved seawater adjusted to treatment temperature and *P*_CO_2__ levels and a *P*_O_2__ of ∼100% air saturation. Six respirometry vials were randomly selected to serve as blanks and were filled with air-saturated seawater for a total of 14 vials per trial: 8 embryo vials (one per replicate tank) and 6 blanks. Embryos were allowed to acclimate to vial conditions for 45 min, after which the seawater in the vial was fully exchanged and the vials were sealed. The trials were conducted within a walk-in environmental chamber where the temperature was maintained at the experimental temperature at time of sampling: 8.7–8.8°C for ambient and 11.0, 12.2 and 13.2°C under the heatwave regime at 6, 7 and 8 dpf, respectively. The respirometry vials were submerged in a water bath maintained at the measurement temperature to reduce possible temperature fluctuations when the walk-in door was opened. Trials were conducted in the dark under low red light to minimize disturbance to the embryos.

Oxygen concentrations (µmol l^−1^) were measured and recorded using a Fibox4 oxygen meter equipped with a PSt3 fiber optic sensor (PreSens) using Presens Measurement Studio (v. 2). The sensor was calibrated prior to each trial with a two-point calibration using air-saturated seawater and a concentrated NaSO_3_ solution (*P*_O_2__=0) adjusted to the trial temperature. After recording initial oxygen concentrations, vials were slowly inverted every 5 min to reduce oxygen stagnation around the embryos. Blank vials were fitted with a small glass bead (4 mm diameter) to promote mixing. Oxygen concentrations were recorded at ∼15 min intervals until levels reached ∼80% air saturation within embryo vials. The duration of trials varied between 71 and 141 min depending on the temperature treatment and age. At the conclusion of the trial, all embryos were recounted and examined for irregularities that would have affected *Ṁ*_O_2_ _(i.e. mortality or hatching), and none were identified. Respirometry vials were filled and shaken with 90% ethanol for sterilization between trials.

Oxygen consumption data were analyzed in R (v. 4.0.2) using Rstudio (v. 1.3.1). Linear regressions between oxygen concentration and time were fitted for each respirometry vial. Regression residuals were analyzed for outlying values and clear outlying measurements were removed from blank vials only. The correlation of determination (*R*^2^) for all fitted regressions for vials containing embryos was >0.925. Derived slopes were used to calculate rates of individual embryo *Ṁ*_O_2__ (nmol O_2_ h^−1^) after accounting for the seawater volume of the respirometry vial (total vial volume−total volume of eggs), background oxygen change (calculated as the mean ΔO_2_ of all blank vials) and the number of embryos per vial. For some trials, the ΔO_2_ of blank vials was slightly positive, but in all cases blank ΔO_2_ was less than 8% of ΔO_2_ in embryo vials. ANOVA indicated that egg surface area was significantly affected by a *P*_CO_2__×temperature interaction (*F*=14.965, *P*<0.001). To account for the influence of egg size, RMR was standardized to the mean embryo surface area of the vial (nmol O_2_ h^−1^ mm^−2^).

#### Hatch morphometrics

Newly hatched larvae were subsampled (*n*=12) on the day of peak hatch (heatwave 11 dpf; ambient temperature 14 dpf) from each replicate tank (192 total). Individual larvae were euthanized via an overdose of MS-222, photographed for analysis of morphometric traits, and dried at 60°C for 24 h. Seven size and shape traits were measured in ImageJ (Fig. S2): standard length (tip of the snout to the end of the notochord; nearest 0.01 mm), somatic body area (total tissue surface area minus the yolk sac and finfold, 0.01 mm^2^), head width (cranial width immediately posterior to the optic disk; 0.01 mm), mean eye width (eye diameter at widest axis, 0.01 mm), post-yolk body depth (dorsal to ventral tissue height including the gut but minus the finfold immediately posterior to the yolk sac; 0.01 mm), post-vent body depth (tissue height immediately posterior to the anus; 0.01 mm) and yolk sac profile area at hatch (0.01 mm^2^). Duplicate dry masses (0.1 mg) were recorded using a micro balance (AT21 Mass Comparator, Mettler Toledo).

After 60 degree-days post-fertilization (ddpf; degree-days=mean incubation temperature×days) at ∼50% development, 10 embryos were sampled from each replicate for measurements of yolk sac surface area at 6 and 7 dpf under heatwave and ambient temperatures, respectively. Replicate mean yolk consumption rates (% day^−1^) were calculated as the percentage change in yolk sac area from 50% development to hatch divided by the incubation interval in days. Embryo growth rate (mm day^−1^) was estimated as the replicate mean standard length at hatch divided by age in dpf at hatch. The energy budget of cleidoic fish egg can be balanced as:
(3)

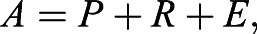
where *A* represents the total energy assimilated from the yolk (i.e. total consumption), *P* is tissue production, *R* is respiration and *E* is excretion (Rombough, 2011). An estimate of production efficiency (i.e. the ratio of yolk consumed to tissues produced) was derived for each replicate as the log of the ratio of larval body area (production, *P*, mm^2^) to the difference in yolk sac area from 50% development to hatch (assimilation, *A*, mm^2^):
(4)

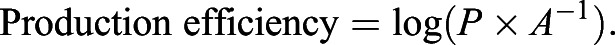


### Statistical analyses

Statistical analyses were performed in R. Main and interactive effects of *P*_CO_2__ level and temperature regime were considered significant at α<0.05. Data are reported as means±1 s.d. unless specified otherwise. Model assumptions were tested using the R package performance (https://CRAN.R-project.org/package=performance). *Post hoc* tests were run using the R package emmeans (https://CRAN.R-project.org/package=emmeans). Plots were produced with ggplot2 (Wickham, 2016). A one-way ANOVA was used to test the effect *P*_CO_2__ on logit-transformed fertilization success. A two-way ANOVA was used to determine *P*_CO_2__×temperature effects on logit-transformed hatching success and time to peak hatch.

A Pearson correlation matrix was constructed to quantify associations between morphometric traits and treatment conditions. A linear dimension reduction was performed on the eight size and shape traits using a principal component analysis (PCA; R package prcomp). The data were centered and scaled prior to PC extraction. Trait correlations, loadings and variance contributions for each PC were calculated in the R package factoextra (https://CRAN.R-project.org/package=factoextra)*.* For each extracted PC, traits with loadings that exceeded ±0.32 were considered practically significant (Tabachnick et al., 2007). Component scores for each PC were assigned to individual larvae. Linear mixed-effect models (LMMs) were constructed to test for significant effects of *P*_CO_2__, temperature regime and their interaction (fixed effects) on PC scores and individual morphometric traits. Tank ID was set as a random intercept to account for the common rearing environment of larvae sampled from the same replicate tank. LMMs were run using the R packages lme4 and lmerTest, or nlme to accommodate variance heterogeneity between groups using the weights and varIdent functions (Bates et al., 2014 preprint; Kuznetsova et al., 2017; http://CRAN.R-project.org/package=nlme). Treatment effects on yolk consumption rate, log-transformed embryo growth rate and production efficiency were tested with two-way ANOVAs.

To evaluate treatment effects on embryo RMR, we first used two-way ANOVAs to test the within-temperature effects of *P*_CO_2__ and embryo age (dpf) on the log-transformed RMR values. Next, two-way ANOVAs tested for significant effects of *P*_CO_2__ and temperature treatment on log-transformed RMR measurements separately for measurement each day. Temperature coefficients (*Q*_10_) for RMR were calculated from embryos at the same developmental stage at 60 ddpf (heatwave: 6 dpf at 11°C; ambient: 7 dpf at 8.8°C) and 70 ddpf (heatwave: 7 dpf at 12.2°C; ambient: 8 dpf at 8.7°C) using the formula:

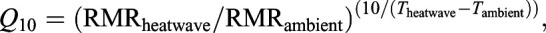
*Q*_10_ coefficients provide a standardized calculation of the change in physiological rates to a 10°C increase in temperature. Additional *Q*_10_ values were calculated for developmental rate, yolk consumption rate and embryo growth rate. For RMR, *T*_heatwave_ and *T*_ambient_ were set to the temperatures used during the respective RMR trials. For other traits, the temperature variables were defined as the average temperature experienced by the embryos during the rearing interval over which the trait was evaluated. *Q*_10_ values were calculated separately for each *P*_CO_2__ level and on combined *P*_CO_2_ _data. To generate confidence intervals (CIs), *Q*_10_ calculations were bootstrapped (*N*=1000) by randomly selecting one replicate value from each temperature treatment and recalculating *Q*_10_. Bias-corrected and accelerated 95% CIs were computed using the R function boot.ci (https://CRAN.R-project.org/package=boot) and *P*-values were calculated from the CIs to quantify significant differences in *Q*_10_ values between *P*_CO_2__ treatments (Altman and Bland, 2011).

## RESULTS

### Fertilization, hatching and survival

**Fig. 2.**
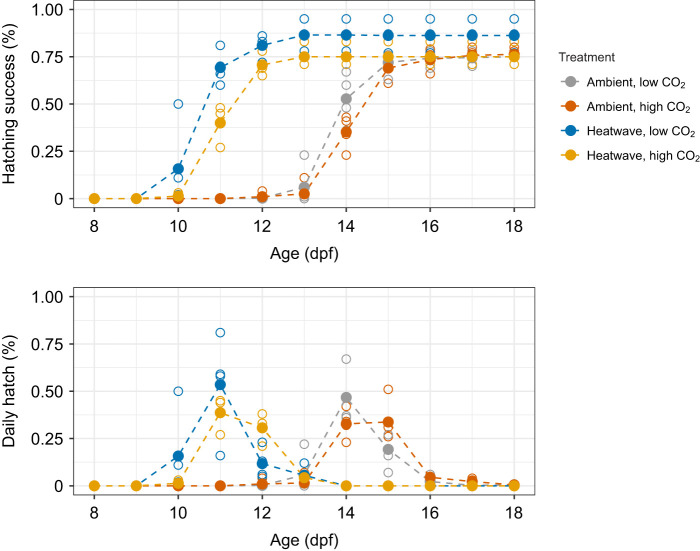
**Hatching timelines for *Clupea pallasii*.** Percent cumulative (upper panel) and daily (lower panel) hatching success under four treatment combinations. Colored dashed lines connecting solid points represent daily mean values while unfilled points denote daily values for individual replicates.

Overall, fertilization success was high (84±7%) and did not significantly vary between *P*_CO_2__ levels (Table 2). Hatching commenced 10 and 13 dpf in the heatwave and ambient temperature treatments, respectively (Fig. 2). Exposure to the heatwave shortened the average time to peak hatch to 11.0±0.5 dpf compared with 14.5±0.5 dpf under ambient temperatures (ANOVA, *P*<0.0001; Table 2, Fig. 2). On average, exposure to high *P*_CO_2__ led to a small but significant delay in hatching by 0.88 days when averaged between temperature treatments (ANOVA, *P*=0.0007; Fig. 2). No interactive effect between *P*_CO_2__ and temperature was detected (Table 2).

**Table 2.**
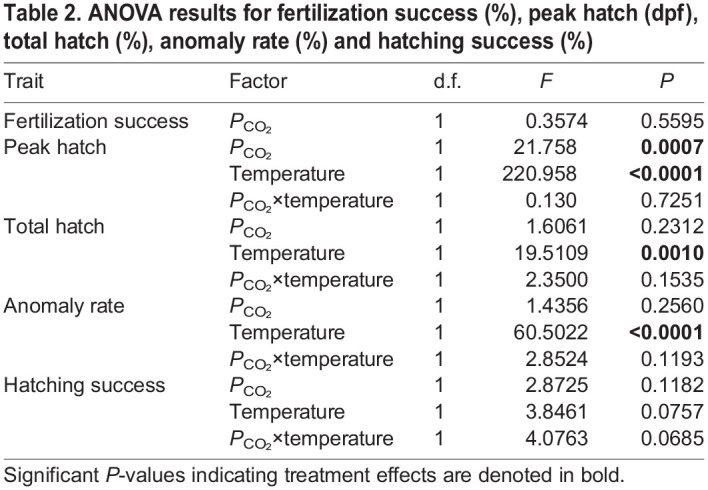
ANOVA results for fertilization success (%), peak hatch (dpf), total hatch (%), anomaly rate (%) and hatching success (%)

**Fig. 3.**
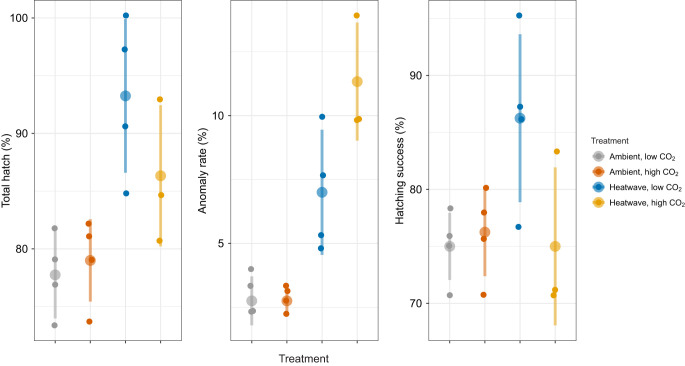
***Clupea pallasii* total hatch (%), anomaly rate (%) and hatching success (%) under four treatment conditions.** Large colored circles with vertical lines represent treatment means±s.d. Small circles show values for individual replicates.

Total hatch was not affected by *P*_CO_2__ level, but it was significantly higher in heatwave replicates (90.3±6.9%) compared with the ambient regime (78.4±3.5%) (ANOVA, *P=*0.001; Table 2, Fig. 3). However, developmental anomalies increased under heatwave conditions by more than three-fold, to 8.9±3.2% (ANOVA, *P*>0.0001; Table 2, Fig. 3). Mean anomaly rates under heatwave conditions were higher under high (11.3±2.3%) compared with low *P*_CO_2__ (7.0±2.4%) but the effect was not significant (Table 2). Higher developmental anomalies under the heatwave regime reduced the average hatching success to 81.4±8.9% when pooled across *P*_CO_2__ treatments, which was not significantly different from ambient replicates (75.6±3.3%) (ANOVA, *P=*0.0757; Table 2, Fig. 3). Owing to a combination of higher total hatch and slightly lower anomaly rate, hatching success in heatwave×low *P*_CO_2__ replicates (86.2±7.4%) was, on average, 11 percentage points higher than the heatwave×high *P*_CO_2__ group (75±6.9%) and both ambient temperature treatments (75–76%; Fig. 3). Despite the difference, the temperature×*P*_CO_2__ interactive effect was not significant (*P*=0.0685, Table 2). Interestingly, mean hatching success under heatwave conditions was 19 percentage points higher than the predicted rate for a static exposure to 13.2°C, the maximum intensity of the heatwave (Fig. S2).

### Hatch morphometrics

**Fig. 4.**
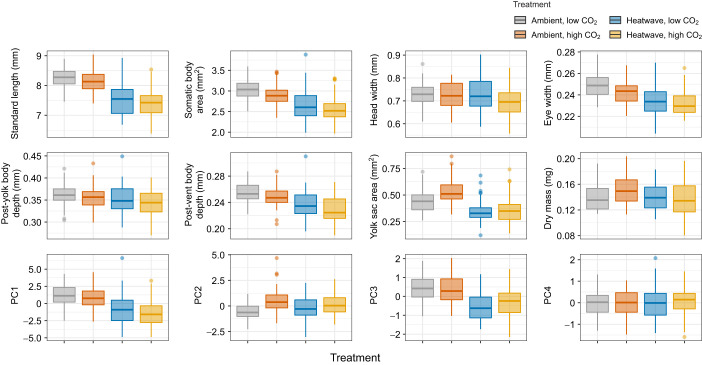
**Morphometric traits and PC scores of *C. pallasii* larvae sampled on the day of peak hatch.** Box plots illustrating distributions of eight morphometric traits and four PC scores from individual larvae reared under four treatment conditions. Boxplots are color coded by treatment condition. The thick horizontal line within each box represents the median value, the box edges represent the 25th and 75th percentiles, vertical lines represent the highest and lowest non-outlying values, and the individual points represent values outside the 95% CI.

In general, morphometric traits were negatively correlated with the heatwave treatment and were weakly associated with *P*_CO_2__ level (Table S2, Fig. 4). The first four extracted PCs accounted for 89.8% of the total variance in morphometric traits and were selected for further analysis. PC1 was identified as the body size component and accounted for the majority of the variance in size and shape (59%). With the exception of yolk sac area and dry mass, all other traits showed significant positive loadings onto PC1 (Table S2). Newly hatched larvae exposed to heatwave conditions exhibited significantly lower PC1 scores, indicating a general reduction in body size at hatch (LMM, *P*<0.001; Table S3), and individually most morphometric traits were significantly reduced under heatwave conditions (Table S3, Fig. 4). When averaged across *P*_CO_2__ treatments, heatwave-exposed hatchlings had a shorter standard length (7.49±0.57 mm) and smaller body area (2.63±0.38 mm^2^) relative to the standard length (8.20±0.33 mm) and body area (2.96±0.26 mm^2^) of larvae from the ambient treatment. PC1 and thus body size was not significantly affected by *P*_CO_2__ level (Table S3).

PC2 accounted for 14.7% of the total variance in morphometric traits and largely captured variation associated with yolk sac area and dry mass (Table S2). PC2 scores were significantly higher under high *P*_CO_2__ conditions (LMM, *P=*0.008; Table S3, Fig. 4). While an interactive effect with temperature was not detected, the effect was more apparent in ambient temperature replicates, where hatchlings exposed to high *P*_CO_2__ were heavier (+10%) and retained larger yolk sacs (+21%) compared with fish from low *P*_CO_2__ treatments (Fig. 4). However, when tested individually in a two-way ANOVA, the effect of *P*_CO_2__ was not significant for either yolk sac area (*P*=0.086) or dry mass (*P=*0.297; Table S3). Thus, PC2 represents the linked variation between yolk sac size and dry mass; that is, hatchlings from high *P*_CO_2__ were slightly heavier because they hatched with larger yolks compared with their low *P*_CO_2__ conspecifics.

PC3 accounted for a small amount of total variance (8.9%) that was positively associated with standard length and eye width, but negatively correlated with head width and post-yolk body depth (Table S1). Heatwave conditions significantly reduced PC3 scores (LMM, *P=*0.001; Table S3, Fig. 4) and PC3 was not affected by *P*_CO_2__ (Table S3, Fig. 4). This indicates that there was a small tendency for heatwave-exposed larvae to develop wider skulls and a deeper body depth while growing to a shorter length with smaller eyes relative to ambient offspring. PC4 account for 6.2% of total variance in morphometric traits and was only associated with variation in eye width (Table S2) and PC4 was unaffected by treatment conditions (Table S2, Fig. 4).

### Treatment effects on physiological rates

**Fig. 5.**
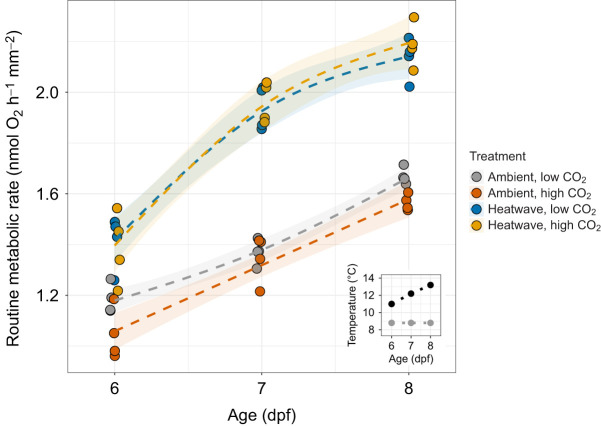
**Embryonic routine metabolic rate (RMR; nmol** **O_2_** **h^−1^** **mm^−2^).** Varying colors denote different treatments. Points represent individual embryo RMR measured from each replicate tank per developmental day. Dotted lines indicate a general additive model fit for each treatment across days and shaded areas denote 1 s.e.m. The inset shows temperature conditions of ambient (gray circles) and heatwave (black circles) treatments for each measurement day.

Under the ambient temperature regime, embryonic RMR increased linearly with developmental day (∼20% day^−1^, ANOVA, *P*<0.001; Table 3, Fig. 5) and exposure to high *P*_CO_2__ significantly reduced RMR by an average of 6.3% across measurement days (ANOVA, *P=*0.006; Table 3, Fig. 5). For embryos exposed to a progressively warming heatwave environment, RMR increased by 39% from 6 to 7 dpf (11 to 12.2°C) (ANOVA, *P<*0.001; Table 3, Fig. 5). From 7 to 8 dpf (12.2° to 13.2°C), RMR again increased significantly (ANOVA, *P<*0.001; Table 3) but the change (+11%) was relatively small compared with the increase observed from 6 to 7 dpf and the average daily increase under ambient conditions. Overall, there was no effect of *P*_CO_2__ in embryos exposed to heatwave conditions. When compared on the same day of sampling, heatwave-exposed embryos had significantly higher RMRs on days 6 (+23%, ANOVA: *P*<0.001), 7 (+44%, ANOVA: *P*<0.001) and 8 (+34%, ANOVA: *P*<0.001) post-fertilization (Table 3, Fig. 5). A significant temperature×*P*_CO_2__ interaction was only detected at 8 dpf (ANOVA, *P*=0.014; Table 3), where high *P*_CO_2__ depressed RMR under ambient but not heatwave conditions (Fig. 5).

**Table 3.**
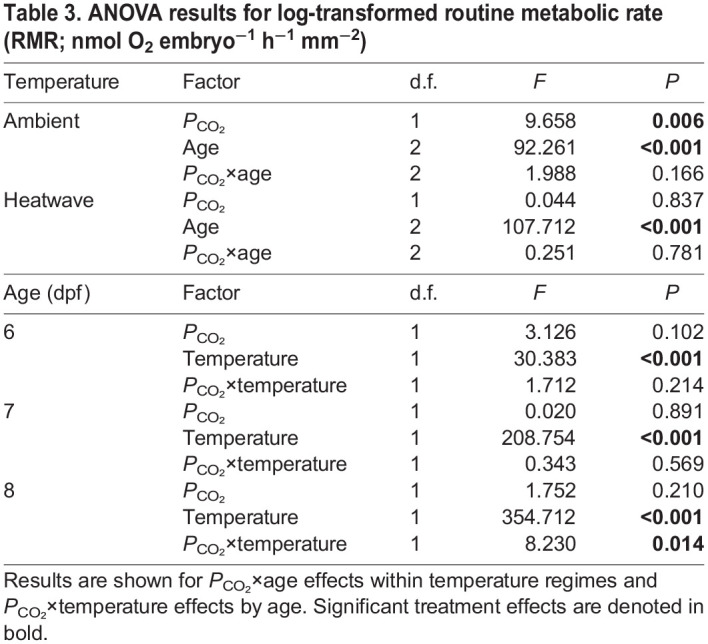
**ANOVA results for log-transformed routine metabolic rate (RMR; nmol** **O_2_** **embryo^−1^** **h^−1^** **mm^−2^)**

**Fig. 6.**
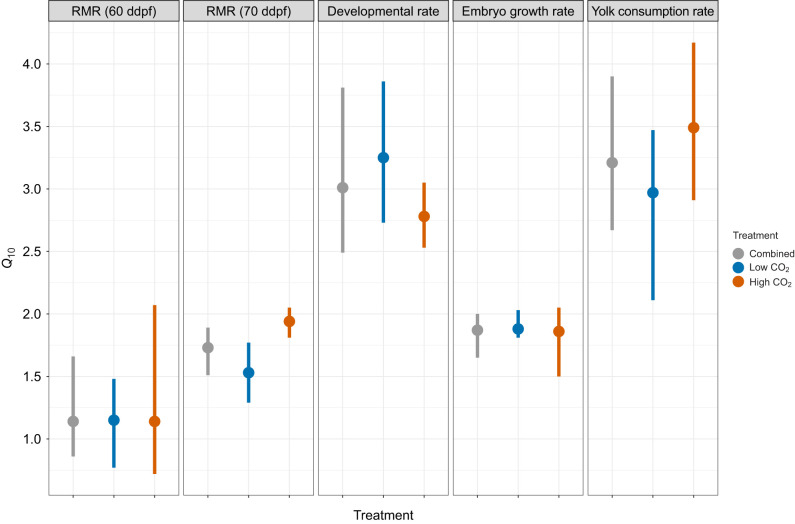
**Summary of *Q*_10_ coefficients by trait.** Mean *Q*_10_ values (circles) ±95% bootstrapped CIs (vertical lines) for embryo RMR (60 and 70 ddpf, nmol O_2_ h^−1^ mm^−2^), developmental rate (% day^−1^), embryo growth rate (mm day^−1^) and yolk consumption rate (% day^−1^).

*Q*_10_ coefficients calculated for embryos at 60 ddpf showed that a heatwave intensity of +2.3°C induced a relatively modest increase in RMR with a mean *Q*_10_ value of 1.15, with little variation between *P*_CO_2__ treatments (Fig. 6). At 70 ddpf, the heatwave intensity increased to +3.5°C above ambient and the mean *Q*_10_ for RMR increased to 1.72 (Fig. 6). Bootstrapped CIs indicated the *Q*_10_ values for 70 ddpf embryos were significantly higher in offspring exposed to high *P*_CO_2__ (s.e.m.=0.20, *Z*=2.01, *P=*0.044). This effect was primarily the result of high *P*_CO_2__ depressing RMR under ambient temperature conditions while under heatwave conditions *P*_CO_2__ level had no effect on RMR (Fig. 5).

**Fig. 7.**
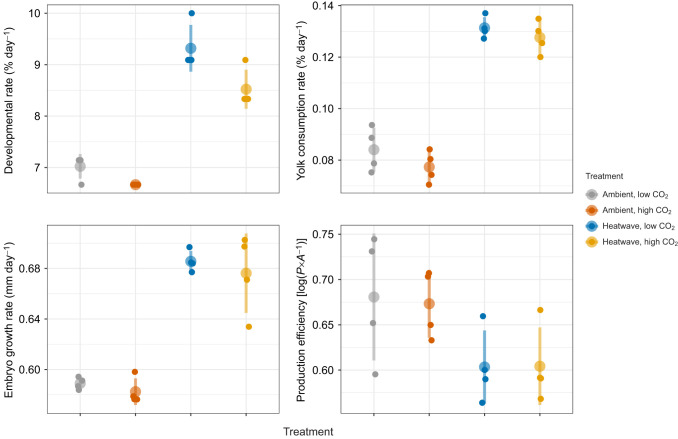
**Developmental rate, yolk consumption rate, embryo growth rate and production efficiency of *C*. *pallasii* under four treatment conditions.** Large colored circles with vertical lines represent treatment means±s.d. Small circles show values for individual replicates.

Developmental rate increased from 6.8±0.2% day^−1^ under ambient temperature conditions to 8.9±0.6% day^−1^ in the heatwave treatments (Fig. 7), which corresponds to an average *Q*_10_ coefficient of 3.01 (Fig. 6). Exposure to high *P*_CO_2__ slowed developmental rate by 0.4% day^−1^ under the ambient temperature and by 0.8% d^−1^ in heatwave regime (Fig. 7). This led to a higher average *Q*_10_ value under low *P*_CO_2_ _conditions, but bootstrapped CIs were not significantly different between *P*_CO_2__ treatments (s.e.m.=0.26, *Z*=1.82, *P=*0.068, Fig. 6)_._ Embryo growth rates were significantly faster under the heatwave (0.68±0.02 mm day^−1^) versus ambient conditions (0.59±0.01 mm day^−1^) (ANOVA, *P<*0.001; Fig. 7, Table S3) which corresponds to a mean *Q*_10_ of 1.87 (Fig. 6). Growth rate was not statistically affected by *P*_CO_2_ _(Table S3).

Heatwave exposure significantly increased yolk consumption rate to 12.9±0.5% day^−1^ relative to a mean of 8.0±0.8% day^−1^ observed under ambient conditions (ANOVA, *P<*0.0001; Table S3, Fig. 7) which corresponded to a mean *Q*_10_ of 3.21 (Fig. 6). This increase in consumption led to a significant reduction (−27%) in the size of remaining yolk reserves at hatch of heatwave offspring compared with ambient conspecifics (LMM, *P*<0.001; Table S3, Fig. 4). Yolk consumption rates were very similar between *P*_CO_2__ treatments under the heatwave, but under the ambient temperature, exposure to high *P*_CO_2__ reduced yolk consumption rate by 9%; however, the effect was not significant (Table S3, Fig. 7). Exposure to the heatwave significantly reduced production efficiency by 10.8%, indicating that heatwave-exposed embryos consumed a greater fraction of yolk reserves to achieve a smaller size at hatch relative to ambient offspring (ANOVA, *P*=0.012; Table S3, Fig. 7). Production efficiency was not influenced by *P*_CO_2__ level (Table S3, Fig. 7).

## DISCUSSION

### Energy metabolism was unaffected by high *P*_CO_2__

We hypothesized that (1) exposure to high *P*_CO_2_ _would increase the aerobic respiration of embryos in order to support the increased biosynthesis of acid–base regulatory factors and (2) this effect would limit the pool of energy available to support higher metabolic rates under heatwave conditions, which would compromise metabolic efficiency and increase mortality. However, after repeatedly measuring *Ṁ*_O_2__ during the onset of heatwave conditions, we found no evidence that exposure to 2300 µatm *P*_CO_2__ increased routine metabolic rates above control *P*_CO_2__ conditions. This lack of CO_2_ effects on metabolism was corroborated by other measured traits: high *P*_CO_2__ did not increase the rate of yolk consumption or reduce production efficiency and size at hatch in either temperature group. We should not discount the possibility that acclimatization to high *P*_CO_2_ _involved subtle molecular changes, such as preferential expression of more efficient regulatory pathways which could not be detected by the methods employed by this study (Kwan et al., 2021). However, regardless of the mechanism, it appears that Pacific herring offspring have a high intrinsic capacity for acid–base regulation that does not require a large energetic investment. Furthermore, this capacity means that high *P*_CO_2__ did not increase embryo mortality by limiting the amount of energy available for thermal acclimation. This finding contrasts with a recent study of Pacific herring in which exposure to ∼1200 µatm *P*_CO_2_ _significantly elevated the rate of embryonic heartbeats by ∼10% at 16°C but not at 10°C (Villalobos et al., 2020). Notably, embryo survival was quite low at 16°C (∼25%), so synergistic effects on metabolic rate in this species might only occur when temperatures approach or exceed upper critical limits, which were not tested in this study (Dahlke et al., 2017).

Significant effects of high *P*_CO_2__ were limited; exposure to 2300 µatm increased time to hatch by ∼1 day in both temperature groups and reduced routine metabolic rate by 6.3% under the ambient temperature regime only. These effects were accompanied by small and non-significant effects of high CO_2_, such as slower growth and yolk consumption, including a 9% reduction in the rate of yolk consumption under the ambient temperature. The mechanism underlying this slight metabolic depression is unclear, as the level of *P*_CO_2__ required to induce an anesthetic response is thought to be orders of magnitude higher than what was applied here (Lefevre, 2019). High *P*_CO_2__ also increased time to hatch in embryos of the northern sand lance, *Ammodytes dubius*, where the delay was thought to be caused by low pH interfering with the hatching process directly (Murray et al., 2019). Regardless of the mechanism, a longer time to hatch is unlikely to be adaptive because it would exacerbate the very high rates of predation inflicted upon Pacific herring eggs (Keeling et al., 2017). However, these small effects should not be overstated and our findings join the bulk of experimental evidence showing that high *P*_CO_2__ conditions consistent with near-future ocean conditions have limited effects (<±10%) on the physiological rates of most marine fish embryos (Flynn et al., 2015; Lefevre, 2019; Leo et al., 2018; Schwemmer et al., 2020).

The effect of high *P*_CO_2__ on embryo mortality was more complex. Hatching success was very similar between *P*_CO_2__ treatments under the ambient regime (75–76%) but in heatwave-exposed replicates it was 11% higher under low (86%) compared with high *P*_CO_2__ (75%). Although the interactive effect was marginally insignificant, the measured difference in survival under the heatwave regime was triggered by a higher total hatch and a lower rate of developmental anomalies under low *P*_CO_2__. Teratogenic effects under high *P*_CO_2__ have been observed in other clupeid early life stages. Exposure to ∼1100 µatm *P*_CO_2__ more than doubled the incidence of malformations in Atlantic herring embryos, with up to 17% of hatchlings showing anomalies when tested across a range of temperatures (Leo et al., 2018). High rates of organ damage were also observed in 3-week-old Atlantic herring larvae reared continuously under ∼2000 µatm *P*_CO_2__ (Frommel et al., 2014). Furthermore, exposure to ∼1200 µatm *P*_CO_2__ increased the rate of mortality by 10% in Pacific herring embryos when simultaneously exposed to 16°C, but anomaly rates were not quantified in that study (Villalobos et al., 2020). Thus, there is accumulating evidence that high *P*_CO_2__ may increase teratogenic effects in clupeid species by impairing normal developmental trajectories. The precise mechanisms involved warrant further examination.

### Heatwave impacts: survival was robust but metabolic efficiency declined

Our analysis of the long-term NERRS data from Padilla Bay, WA, found that nearshore heatwaves can occur during all months of the year and therefore have the potential to impact herring spawning events throughout the reproductive season. Although most years of the time series exhibited just one or two events, the period covering the 2014–2016 North Pacific Heatwave Event showed a spike in activity with 26 nearshore events occurring over just 3 years (see Supplementary Materials and Methods). Seven of top 10 most extreme heatwaves were detected during this period. We modeled the simulated heatwave of the experiment after these extreme events with the expectation that embryo survival would decline as temperatures reached maximum intensity, based on an established thermal reaction norm derived from static-temperature experiments on Salish Sea herring (Alderdice and Velsen, 1971). Contrary to our expectation, heatwave exposure actually increased total hatch counts, but the simultaneous increase to the rate of developmental anomalies acted to reduce overall hatching success to a rate similar to that observed in the ambient temperature regime.

The observed higher-than-expected survival was likely due to the specific timing of the thermal challenge: the heatwave was initiated after embryos had developed under ambient conditions for ∼3.5 dpf and maximum intensity was reached when embryos had attained 75% of full development. This suggests the Pacific herring, like other temperate and cold-water fish, are most stenothermal during the earliest phases of embryogenesis (i.e. during gastrulation) and limits of thermal tolerance expand throughout organogenesis (Dahlke et al., 2020a; Flynn and Todgham, 2018; Mueller et al., 2015). As such, Pacific herring embryos were able to outgrow the lethal effects of our simulated heatwave despite being sourced from a population that reproduces in February and early March, when daily mean temperatures are relatively cold and diel variability is low (8.1±1.2°C) (Sandell et al., 2019). These results suggest that winter-spawning Salish Sea herring retain sufficient thermal plasticity to survive a relatively extreme heatwave event, but whether earlier embryonic stages are more sensitive to the thermal stress of a developing heatwave requires further examination.

The heatwave affected most measured physiological rates, but to varying degrees. For example, rate of development to hatch (*Q*_10_=3.01) was not matched by a proportional increase in embryonic growth rate (*Q*_10_=1.87). This means that despite a faster growth rate, larvae from the heatwave regime were ∼10% smaller at hatch than their conspecifics reared at ambient temperatures. This is consistent with previous findings from Pacific herring (Alderdice and Velsen, 1971; Dinnel et al., 2010; Villalobos et al., 2020) and fits within the broader pattern exhibited by temperate fish of an inverse relationship between hatch size and temperature (Rombough, 1997). It is also assumed that temperature has proportionally equal effect on the rates of growth and yolk consumption, such that production efficiency (i.e. the ratio of tissue produced to yolk assimilated) tends to be insensitive to temperature change within a species' thermal envelope (Houde, 1989; Rombough, 2011). This is a key metabolic relationship, as it enables embryos to achieve exceptionally high specific growth rates via the efficient conversion of maternally derived yolk reserves into new embryonic tissue (Kamler, 2008; Rombough, 2006).

However, we found that heatwave-exposed embryos exhibited a 10.8% reduction in production efficiency as the *Q*_10_ coefficient for yolk consumption (*Q*_10_=3.21) and was substantially greater than the estimate for growth rate. In effect, this meant that heatwave-exposed embryos consumed a larger fraction of their yolk supply to achieve a smaller body size at hatch. This finding is somewhat contradictory with the modest *Q*_10_ coefficients we estimated for RMR during the onset of the heatwave (*Q*_10_=1.17 at +2.3°C and *Q*_10_=1.70 at +3.5°C). We would have expected a greater effect on RMR to induce a loss of production efficiency, particularly given that fish embryos typically exhibit *Q*_10_ coefficients close to 3.0 (Rombough, 2011). However, we could not assess the *Q*_10_ for RMR at maximum heatwave intensity (+4.4°C) as the handling required to make the measurements induced hatching in late-stage embryos, but it is probable that it continued to increase as *Q*_10_ values for oxygen demand tend to closely align with yolk utilization rates (Houde, 1989; Rombough, 2011). This is consistent with findings from the lake whitefish (*Coregonus clupeaformis*), where cost of development (which encompasses all energetic processes) showed the largest increase with temperature during the ladder stages of embryogenesis (Mueller et al., 2015).

The higher incidence of developmental anomalies under the heatwave conditions suggests that some embryos were suffering cellular damage related to thermal stress (Basu et al., 2002). Thus, the observed production inefficiencies may be related to the energetically intensive processes related to countering heat stress. As standard metabolic rates increase with warming, additional energetic costs accumulate with the increased biosynthesis of heat shock proteins (Alfonso et al., 2021; Takle et al., 2005). A metabolic trade-off to preserve cellular integrity at the expense of growth could negatively influence recruitment dynamics because higher rates of predation and starvation are inversely correlated with larval size (Hendry et al., 2001; Miller et al., 1988, but see Pepin, 1991). Furthermore, lingering growth deficiencies induced during embryogenesis could be exacerbated during larval and juvenile growth owing to large-scale ecosystem disruption and changes in predator and prey dynamics that accompany prolonged heatwave events (von Biela et al., 2019). Particularly at risk is the Cherry Point herring population, which spawns later than all other herring stocks in the Salish Sea, reaching peak activity in mid-May (Sandell et al., 2019), when nearshore temperatures are warmer and more variable (13.5±2.6°C) compared with conditions for winter spawners and the risk for high intensity heatwaves appears to be greatest in late spring. Despite this late spawning phenology, a recent study on the genetic diversity of Salish Sea herring found no evidence for thermal adaptation in the Cherry Point stock (Petrou et al., 2021), suggesting that embryos are already developing near their maximal thermal limits. The Cherry Point stock was once the largest in the region but has suffered severe declines in recent years (Sandell et al., 2019). Rising ocean temperatures and the emergence of intense regional heatwaves may now be constraining the reproductive viability of this unique population.

### Conclusions and future directions

We found that Pacific herring embryos could successfully acclimate to the stimulated heatwave without a reduction in survival, even when simultaneously exposed to high *P*_CO_2__ conditions. The effects of high *P*_CO_2__ were minimal and we found no evidence that acidification increased aerobic respiration or increased energy expenditure towards acid–base regulation. However, the heatwave did elicit measurable metabolic trade-offs, including a significant reduction in the production efficiency of developing embryos, resulting in a smaller size at hatch with smaller yolk reserves. Quantifying how these effects will impact the fitness of wild offspring will require long-term studies that can properly assess carryover effects to later life stages. Furthermore, these experimental data need to be synthesized with field observations of community-level heatwave responses to understand the dynamic effects on the predators and prey of herring larvae.

There is a growing understanding that fluctuating environments elicit different physiological responses compared with static environments with the same average values (Cross et al., 2019; Schunter et al., 2021; Tomasetti et al., 2021), necessitating that environmental variability be incorporated into experimental designs (Kroeker et al., 2020; Morash et al., 2018). Although the present study provides critical data on the influence of a developing heatwave, our experimental treatments did not simulate the full complexity of nearshore habitats. For example, high-frequency variations of temperature and *P*_CO_2__ that fluctuate over diel and tidal cycles are ubiquitous in shallow coastal marine systems (Baumann and Smith, 2017; Pacella et al., 2018). Extreme heatwaves that increase daily mean temperatures will also increase the amplitude of these fluctuations and extend the duration of extreme conditions (Schlegel et al., 2017; Wetz and Yoskowitz, 2013; Zhang et al., 2013). Biological proofs of Jensen's inequality tell us that organismal performance under variable environments is lower than under static conditions of the same mean, and that performance continues to decline as variability increases (Denny, 2017; Morash et al., 2018). Therefore, it is possible that the outcomes of this study could be different if high-frequency fluctuations of temperature and *P*_CO_2__ were superimposed on top of the daily heatwave progression. This could be addressed by future investigations specifically designed to assess the phenotypic effects of high-frequency exposures to extreme conditions.

## Supplementary Material

Supplementary information
