# Supplementary material for: High PCO2 does not alter the thermal plasticity of developing Pacific herring embryos during a marine heatwave
Source: J Exp Biol. 2022 Mar 10;225(5):jeb243501. doi: 10.1242/jeb.243501 (PMC8976945; doi:10.1242/jeb.243501)
Supplement: Supplementary information [file jexbio-225-243501-s1.pdf]

Supplementary Materials & Methods.

Heatwave Analysis

Methods: Heatwave dynamics (i.e., duration, rate of onset, and maximum intensity) were determined by analyzing an 18-year temperature dataset (2002-2020) from the NERRS Ploeg Channel monitoring station in Padilla Bay, WA (48°33'22.8"N 122°31'51.2"W). The station code is PDBBPWQ and the dataset was downloaded from the NERRS Centralized Data Management Office (https://cdmo.baruch.sc.edu/dges/). The R package heatwaveR (Schlegel and Smit, 2018) was used to identify heatwaves in the timeseries based on a selection criteria of daily mean temperatures exceeding the 90th percentile of the long-term seasonal climatology for a minimum of 5 consecutive days (Hobday et al., 2016).

Results: A total of 45 events were detected in the 18-year dataset. Heatwaves occurred during all months at a roughly equal frequency between seasons (11 or 12 per season). Maximum intensity ranged from 1.67° - 5.33°C above seasonal climatology, with a mean (±SD) of 2.97±0.79°C. However, there was a clear seasonal fluctuation in maximum heatwave intensity as the strongest events occurred in late-spring and summer. The most extreme event occurred in June 2014. It reached a maximum intensity of 5.32°C with a rate of onset of 0.56 °C×d-1 and lasted for eight consecutive days (Table S1). Event duration ranged from 5 to 20 d (mean 7.3±3.1 d) while rate of onset ranged between 0.08° C × d-1 to 1.01°C × d-1 (mean 0.46±0.22°C × d-1). There was a weak relationship between maximum intensity and rate of onset. More intense heatwaves tended to manifest more quickly, but there were several outlying events (linear regression, p = 0.00289, r2 = 0.1696). There was no relationship between maximum intensity and event duration. However, there was a weak negative relationship between rate of onset and duration, such that heatwaves that manifested quickly had shorter durations (linear regression, p = 0.0087, r2 = 0.1297). Again, there were clear outlying events to this trend. The period around the 2014-2016 North Pacific Heatwave produced more heatwaves and the most intense events compared to any other period in the timeseries (2014: 7 events, 2015: 12 events, 2016: 7 events, Fig. S1). Most other years experienced just 1-2 events, but a smaller heatwave outbreak occurred during 2003-2005 with 3 – 4 events per year (Fig. S1). Detailed characteristics of the top 10 heatwave events are listed in Table S1.

Journal of Experimental Biology • Supplementary information

Converting pH_{NIST} to pH_T

Methods: To reduce the error associated with seawater pH measurements calibrated on the NIST scale (Dickson et al., 2015), an extensive cross-calibration was made between the handheld Orion electrode and the diode array spectrophotometer to produce a robust probe-specific linear relationship to convert pH_{NIST} values measured with the handheld to pH_T. A total of 36 seawater sample will prepared by stripping or adding small amounts of dissolved CO_2 to seawater to achieve pH levels across the range of pH values recorded during the experiment. All measurements were made room temperature (20-23°C) and a salinity of 32. After vigorously mixing the seawater samples, pH_{NIST} was recorded with the handheld electrode and then the sample was immediately injected into the jacketed cuvette and pHT was measured with the diode array spectrophotometer (Love et al., 2017). A conversion formula was derived by fitting the relationship with a linear model (pHT = 0.91344 × pHNIST + 0.59905, $R^2 > 0.99$)

Journal of Experimental Biology • Supplementary information

Table S1. Summary of the top 10 heatwave events detected at the Ploeg Channel Monitoring Station in Padilla Bay, WA from 2002-2020. Cumulative intensity is sum of daily positive temperature anomaly for the duration of the heatwave.

Rank	Duration (days)	Start	Peak	Max intensity (°C)	Cum. Intensity (°C)	Rate of onset (°C d^{-1})
1	8	6/3/2014	6/8/2014	5.32	30.82	0.56
2	5	4/19/2016	4/22/2016	4.71	17.21	0.67
3	6	6/8/2015	6/11/2015	4.64	21.24	0.65
4	10	6/23/2015	6/26/2015	4.03	34.24	0.33
5	7	7/20/2003	7/23/2003	4.01	18.86	0.61
6	6	7/23/2004	7/25/2004	3.96	16.74	0.79
7	6	5/26/2015	5/30/2015	3.93	18.58	0.53
8	5	4/30/2016	5/3/2016	3.87	13.33	0.57
9	6	7/31/2014	8/4/2014	3.78	16.18	0.54
10	13	3/30/2016	4/1/2016	3.56	32.16	0.84

Journal of Experimental Biology • Supplementary information

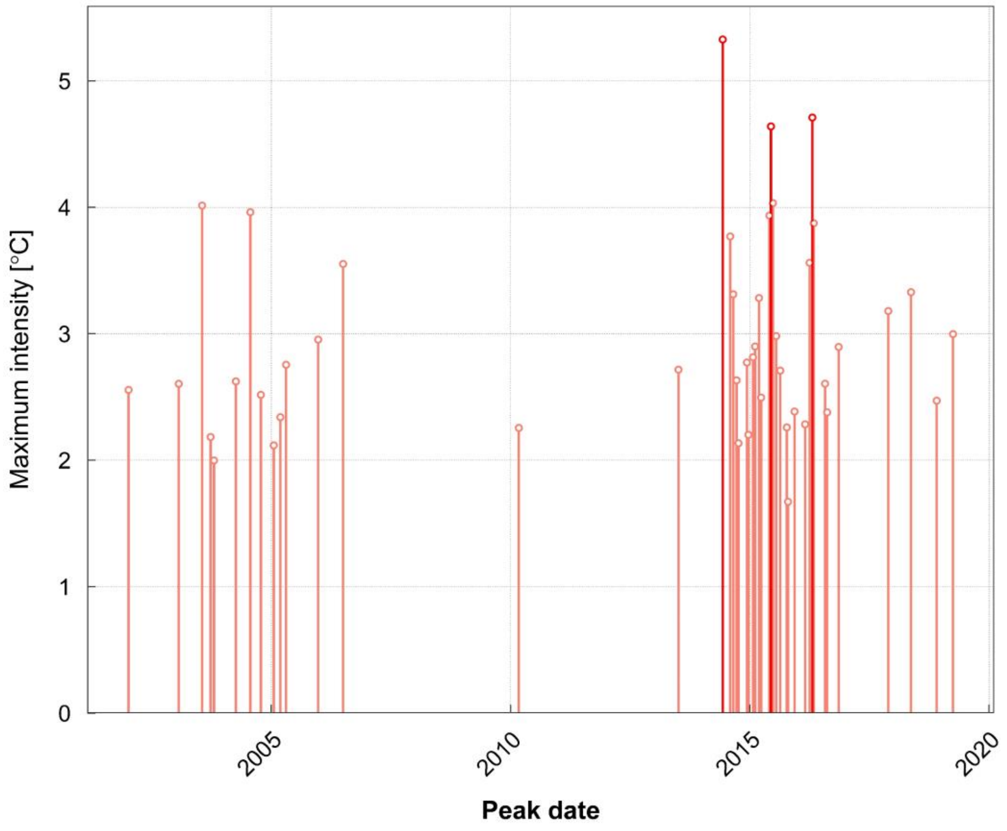

Fig. S1. A lolli-plot of all detected heatwave events. The vertical red lines denote the maximum intensity (°C) of each event.

Journal of Experimental Biology • Supplementary information

References

Dickson, A. G., Camões, M. F., Spitzer, P., Fisicaro, P., Stoica, D., Pawlowicz, R. and Feistel, R. (2015). Metrological challenges for measurements of key climatological observables. Part 3: seawater pH. *Metrologia* **53**, R26-R39.

Hobday, A. J., Alexander, L. V., Perkins, S. E., Smale, D. A., Straub, S. C., Oliver, E. C. J., Benthuysen, J. A., Burrows, M. T., Donat, M. G., Feng, M. et al. (2016). A hierarchical approach to defining marine heatwaves. *Progress in Oceanography* **141**, 227-238.

Love, B. A., Olson, M. B. and Wuori, T. (2017). Technical Note: A minimally invasive experimental system for pCO2 manipulation in plankton cultures using passive gas exchange (atmospheric carbon control simulator). *Biogeosciences* **14**, 2675-2684.

Schlegel, R. and Smit, A. (2018). heatwaveR: A central algorithm for the detection of heatwaves and cold-spells. *Journal of Open Source Software* **3**, 821.

Table S2. Pearson correlation coefficients matrix of treatment conditions, morphometric traits, and PC 1-4 scores. Traits are abbreviated as standard length (SL), somatic body area (SBA), head width (HW), eye width (EW), post-yolk body depth (PYBD), post-vent body depth (PVBD), yolk-sac area (YSA), and dry weight (DW).

	**Temp**	***p*CO_2**	**SL**	**SBA**	**HW**	**EW**	**PYBD**	**PVBD**	**YSA**	**DW**	**PC1**	**PC2**	**PC3**	**PC4**
**Temp**	-	-	-0.628	-0.461	-0.135	-0.431	-0.16	-0.454	-0.472	-0.177	-0.482	0.004	-0.478	0.065
***p*CO_2**	-	-	-0.086	-0.146	-0.148	-0.166	-0.121	-0.174	0.194	0.144	-0.101	0.327	0.076	-0.014
**SL**	-0.628	-0.086	-	0.906	0.536	0.608	0.487	0.725	0.463	0.403	0.858	-0.134	0.309	-0.224
**SBA**	-0.461	-0.146	0.906	-	0.733	0.626	0.72	0.81	0.435	0.431	0.948	-0.181	0.001	-0.161
**HW**	-0.135	-0.148	0.536	0.733	-	0.481	0.687	0.55	0.359	0.322	0.776	-0.139	-0.435	0.201
**EW**	-0.431	-0.166	0.608	0.626	0.481	-	0.403	0.567	0.332	0.207	0.697	-0.293	0.374	0.497
**PYBD**	-0.16	-0.121	0.487	0.72	0.687	0.403	-	0.574	0.436	0.412	0.778	0.022	-0.501	0.042
**PVBD**	-0.454	-0.174	0.725	0.81	0.55	0.567	0.574	-	0.315	0.305	0.816	-0.287	0.099	-0.285
**YSA**	-0.472	0.194	0.463	0.435	0.359	0.332	0.436	0.315	-	0.679	0.621	0.652	0.147	0.181
**DW**	-0.177	0.144	0.403	0.431	0.322	0.207	0.412	0.305	0.679	-	0.576	0.714	0.048	-0.122
**PC1**	-0.482	-0.101	0.858	0.948	0.776	0.697	0.778	0.816	0.621	0.576	-	-	-	-
**PC2**	0.004	0.327	-0.134	-0.181	-0.139	-0.293	0.022	-0.287	0.652	0.714	-	-	-	-
**PC3**	-0.478	0.076	0.309	0.001	-0.435	0.374	-0.501	0.099	0.147	0.048	-	-	-	-
**PC4**	0.065	-0.014	-0.224	-0.161	0.201	0.497	0.042	-0.285	0.181	-0.122	-	-	-	-

Journal of Experimental Biology • Supplementary information

Table S3. Summary statistics for LMMs and ANOVAs testing growth traits. Results for LMMs testing individual PC scores and morphometric traits. Numerator degrees of freedom (NumDF) and denomination degrees of freedom (DenDF) are presented. ANOVA results are given for yolk consumption rate (YCR), embryo growth rate (GR), and production efficiency (PE). Significant test results are denoted in bold.

**LMMs**					
**Trait**	**Factor**	**NumDF**	**DenDF**	***F***	***p***
PC1	*p*CO_2	1	12	1.347	0.268
	Temp	1	12	22.709	**<0.001**
	pCO_2 × temp	1	12	0.212	0.654
PC2	*p*CO_2	1	12	10.270	**0.008**
	Temp	1	12	0.424	0.527
	pCO_2 × temp	1	12	1.919	0.191
PC3	*p*CO_2	1	12	0.452	0.514
	Temp	1	12	17.423	**0.001**
	pCO_2 × temp	1	12	0.907	0.360
PC4	*p*CO_2	1	12	0.017	0.898
	Temp	1	12	0.362	0.559
	pCO_2 × temp	1	12	0.125	0.730
SL	*p*CO_2	1	12	3.442	0.088
	Temp	1	12	54.230	**<0.001**
	pCO_2 × temp	1	12	0.037	0.851
SBA	*p*CO_2	1	12	3.313	0.094
	Temp	1	12	21.062	**<0.001**
	pCO_2 × temp	1	12	0.001	0.987
HW	*p*CO_2	1	12	1.269	0.282
	Temp	1	12	1.401	0.259
	pCO_2 × temp	1	12	0.751	0.403
EW	*p*CO_2	1	12	3.422	0.089
	Temp	1	12	22.948	**<0.001**
	pCO_2 × temp	1	12	1.238	0.288
PYBD	*p*CO_2	1	12	0.945	0.350
	Temp	1	12	1.646	0.224
	pCO_2 × temp	1	12	0.180	0.679
PVBD	*p*CO_2	1	12	5.599	**0.036**
	Temp	1	12	38.016	**<0.001**
	pCO_2 × temp	1	12	0.004	0.952
YSA	*p*CO_2	1	12	3.552	0.084
	Temp	1	12	20.8990	**<0.001**
	pCO_2 × temp	1	12	2.071	0.176
DW	*p*CO_2	1	12	1.191	0.297
	Temp	1	12	1.803	0.204
	pCO_2 × temp	1	12	1.091	0.317
**ANOVAs**					
**Trait**	**Factor**	**DF**	**F**	***p***	
YCR	*p*CO_2	1	0.556	0.133	
	Temp	1	70.500	**<0.0001**	
	pCO_2 × temp	1	0.102	0.653	
GR	*p*CO_2	1	0.984	0.341	
	Temp	1	131.732	**<0.0001**	
	pCO_2 × temp	1	0.016	0.902	
PE	*p*CO_2	1	0.018	0.896	
	Temp	1	8.761	**0.012**	
	pCO_2 × temp	1	0.028	0869	

Journal of Experimental Biology • Supplementary information

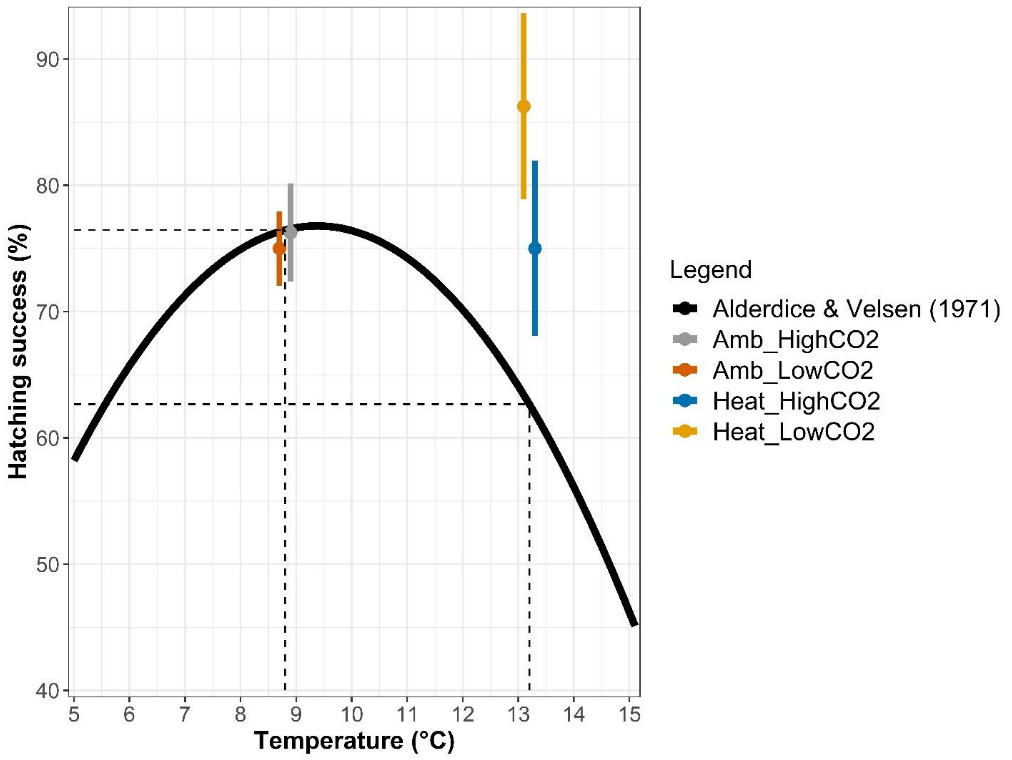

Fig. S2. The thermal reaction norm for embryo survival in Pacific herring. Colored circles denote the average hatching success ± s.d. under the four treatment conditions tested in this study. The solid black curve shows the temperature reaction norm for embryo survival (%) under static temperatures predicted by Alderdice & Velsen (1971) at salinity of 32. Dashed lines connect the predicted survival rates for the ambient temperature regime (~8.8°C) and maximum heatwave intensity (~13.2°C).

Journal of Experimental Biology • Supplementary information

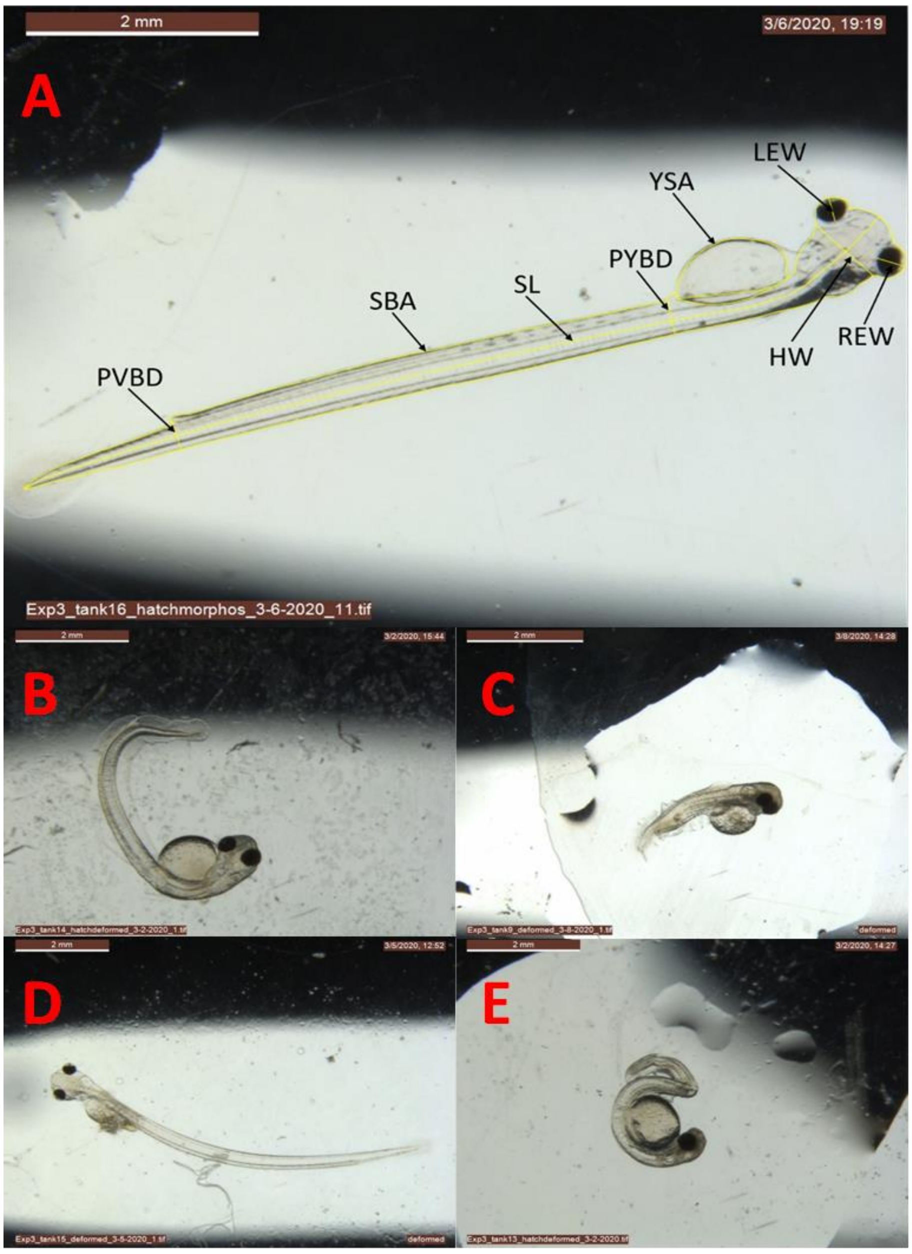

Fig. S3. A. Schematic of the eight morphometric measurements made on healthy hatchings; PVBD (post-vent body depth), SBA (somatic body area), SL (standard length), PYBD (post-yolk body depth), YSA (yolk sac area), HW (head width), LEW (left eye width), REW (right eye width). **B.** Developmental anomaly: notochord curvature **C.** Developmental anomaly: notochord shortening and underdeveloped jaw. **D.** Developmental anomaly: yolk sac edema. **E.** Developmental anomaly: notochord twist and underdeveloped jaw.
